# Herbal Mixture of *Carthamus tinctorius* L. Seed and *Taraxacum coreanum* Attenuates Amyloid Beta-Induced Cognitive Dysfunction In Vivo

**DOI:** 10.3390/foods11020142

**Published:** 2022-01-06

**Authors:** Meitong He, Jihyun Kim, Chanhum Park, Eunju Cho

**Affiliations:** 1 Department of Food Science and Nutrition & Kimchi Research Institute, Pusan National University, Busan 46241, Korea; skyham16@gmail.com; 2 Department of Food Science, Gyeongsang National University, Jinju 52725, Korea; jihyunkim@gnu.ac.kr; 3 Institute of New Frontier Research Team, Hallym Clinical and Translational Science Institute, Hallym University, Chuncheon 24252, Korea; ptman123@naver.com

**Keywords:** cognitive dysfunction, amyloidogenesis, *Carthamus tinctorius* L. seed, *Taraxacum coreanum*

## Abstract

Deposition of amyloid-beta (Aβ) in the aging brain has been often observed and is thought to be a pathological feature of Alzheimer’s disease. The use of natural products for disease prevention and treatment is gaining attention worldwide. *Carthamus tinctorius* L. seed and *Taraxacum coreanum* have been used as traditional medicines in Asian countries, where they have been reported to exert anti-inflammatory and anti-oxidative effects. It has been demonstrated that the combination of *C. tinctorius* L. seed and *T. coreanum* has an effect on cognitive enhancement, indicating a ratio of 5:5 synergistically enhancing learning and memory abilities in comparison with a single treatment. Here, we aimed to investigate the protective effect of *C. tinctorius* L. seed and *T. coreanum* mixture (CT) at different concentrations on cognition in Aβ_25-35_-infused mice. CT-administered mice showed significant cognitive improvement in the T-maze, novel object recognition, and Morris water maze tests. Moreover, amyloidogenesis-related proteins, such as β-secretase and γ-secretase, were detected and their protein levels decreased after treatment with CT. Our study shows that CT attenuates cognitive dysfunction by improving learning and memory capability and regulating Aβ-related proteins in Aβ_25-35_-injected mice. These findings suggest that CT might be a candidate for functional food on cognitive improvement.

## 1. Introduction

Alzheimer’s disease (AD) is the most common type of dementia, with neuropathological features consisting of amyloid plaque deposition, neurofibrillary tangle formation, and synaptic damage [1,2]. AD is characterized by cognitive and memory decline with age, ultimately leading to death [2]. Since the pathogenesis of AD and drugs for treatment of AD are not clearly established, research on AD prevention is a considerable concern as a public health issue. According to the “amyloid cascade hypothesis” by Hardy and Higgins [1], amyloid beta (Aβ) peptides are the main constituents of amyloid plaques, and their deposition is an initial event in AD pathology. An “amyloidogenic pathway” indicates that Aβ peptides derived from mutations of amyloid precursor protein (APP) are produced by cleavage of β-secretase and γ-secretase [3,4]. Aβ peptides can disrupt cellular ionic balance, activate inflammatory responses, induce oxidative stress, promote apoptosis, and cause neurotransmitter deficits, which ultimately lead to AD [5]. A frequently used model to understand the pathology of AD involves injecting Aβ peptides into the brain. Activated microglial cells have been observed with intracerebroventricular (ICV) injection of Aβ peptides into mouse brain [6].

Over the last two decades, various experimental and clinical therapies have been used to treat AD [7]. Indeed, some drugs, such as acetylcholinesterase inhibitors (donepezil, rivastigmine, etc.) and N-methyl D-aspartate receptor antagonists (memantine), have been approved [8]. However, these drugs can provide only symptomatic relief for a short period and are accompanied by serious side effects [9]. For example, the most frequent adverse effects of donepezil are nausea, dizziness, and muscle cramps [10]. Therefore, scientists have devoted efforts to find new materials with low side effects. Recently, natural materials that are safer and more easily accessible from nature have attracted the attention of researchers [7]. 

*C. tinctorius* L. is a popular plant used for treatment of diseases in Asian countries [11]. In particular, seeds from *C. tinctorius* L. have been traditionally used to treat bone disease [12]. Recently, *C. tinctorius* L. seed showed bioactive effects on anti-inflammation, anti-adipogenesis, and anti-oxidation [13,14,15]. On the other hand, *T. coreanum* has been reported to exert various biological activities, such as antioxidant, anti-inflammatory, antibacterial, and anti-cancer activity [16,17,18]. Several studies have demonstrated that the ethyl acetate fraction of *T. coreanum* inhibits hydrogen peroxide-induced oxidative stress in vitro [19] and protects memory and cognitive function in Aβ peptide-injected mice [20]. 

The synergistic effect of the combination of *C. tinctorius* L. seed and *T. coreanum* at different ratios has been reported. Previous results show that cognitive function synergistically improved after administration of the combination at a ratio of 2:8, 5:5, and 8:2, compared to the single administered group. In particular, APP processing-related protein expression was significantly downregulated and insulin-degrading enzyme expression was upregulated at a ratio of 5:5 [21]. Based on our previous results, in the present study, we aimed to understand whether using a mixture of *C. tinctorius* L. seed and *T. coreanum* (CT) at a 1:1 ratio at different concentrations improves Aβ-induced cognitive dysfunction in an AD mouse model. We explored Aβ-induced memory and cognitive dysfunction using a series of behavioral tests and investigated the molecular mechanism of the amyloidogenic pathway in an Aβ-induced AD mouse model. 

## 2. Materials and Methods

### 2.1. Sample Preparation

The samples were obtained from the Department of Medicinal Crop Research (National Institute of Horticultural and Herbal Science, Rural Development Administration, Eumseong, Korea). Briefly, dried *C. tinctorius* L. seeds and *T. coreanum* were mixed at a ratio of 1:1 and extracted with water at 90 °C for 8 h. Subsequently, the extracts were concentrated at 60 °C and freeze-dried for 48 h. The water extract of CT at a 1:1 ratio was obtained by dissolving it in water before the experiments.

### 2.2. Aβ Peptide Preparation and Injection

Aβ_25-35_ peptides (Sigma-Aldrich, St. Louis, MO, USA) were dissolved in 0.9% NaCl solution to obtain a final concentration of 5 nM and incubated at 37 °C for 72 h to aggregate [22]. The ICV injection was administered according to the Franklin and Paxinos atlas [23]. Mice were anesthetized in advance using a solution containing a mixture of Zoletil 50^®^ (30 mg/kg) and Rompun (10 mg/kg). The mice were administered ICV injection using a stereotaxic apparatus with coordinates of −0.2 mm (anteroposterior), −1.0 mm (mediolateral), and −2.5 mm (dorsoventral) [24]. The total injection volume per mouse was 5 µL and the needle was retained at the injection site for 2 min. The normal group was injected with 0.9% NaCl solution. After injection, the mice were returned to their cages for recovery.

### 2.3. Animals and Treatment

Five-week-old male ICR mice weighing 29–31 g were purchased from OrientBio (Seongnam, Korea). All mice were randomly divided into groups (*n* = 8) and housed in cages with access to food and water. The feeding environment was maintained with a 12/12 h light–dark cycle at a temperature of 20 ± 2 °C and humidity of 50 ± 10%. The mice were acclimated for one week before the experiments. All animal care procedures, including experiments, were strictly conducted according to the guidelines of the Pusan National University Institutional Animal Care and Use Committee (PNU-IACUC, approval number: PNU-2019-2145).

**Figure 1:**
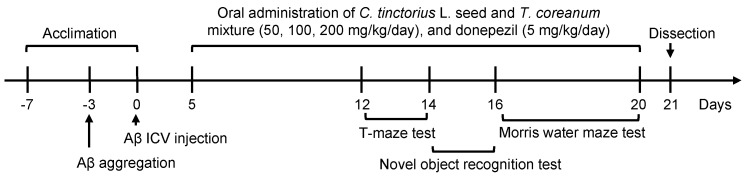
Experimental design.

The normal group was administered 0.9% NaCl solution by ICV injection and orally administered water. The control group was administered Aβ_25-35_ peptides by ICV injection and orally administered water. The sample groups were administered with Aβ_25-35_ peptides by ICV injection and orally administered with CT at 50, 100, and 200 mg/kg. The positive control (DO) group was treated with ICV injection with Aβ_25-35_ peptides and orally administered donepezil (Sigma–Aldrich, St. Louis, MO, USA) at 5 mg/kg. CT and donepezil extracts were dissolved in water. Oral administration was initiated 3 days after ICV injection and was continued for 14 days. The schedule of Aβ_25-35_ peptide injection and behavioral tests are shown in Figure 1.

### 2.4. T-Maze Test

The T-maze is a T-shaped apparatus painted in acrylic black color, consisting of three arms (labeled left, right, and starting point). The test was performed for 2 days. On the first day, the right arm was blocked by a baffle. The mice were probed from the starting point to the left arm and the number of mice that entered the left arm was recorded within 10 min. Mice were then placed in cages and rested for 24 h. On the second day, both the left (old route) and right (new route) arms were opened. Mice were allowed to explore the two arms for 10 min, and the number of arm entries was recorded. The spatial cognitive ability (%) was calculated as follows: [new route or old route/(old route + new route)] × 100 [25].

### 2.5. Novel Object Recognition Test

A novel object recognition test was used to investigate the object recognition ability. In the test, two entirely identical objects (labeled A and A′) and another object entirely different from the first two objects (B) were used. The test was performed using an open-square black box. On the first day, objects A and A’ were fixed in the middle of the box with some distance between them. Mice were allowed to move and explore the objects for 10 min, and the number of times the objects were touched was recorded. Next, the mice were placed in cages and rested for 24 h. The following day, object A’ was replaced by object B, and the mice were allowed to explore A (familiar object) and B (novel object) for 10 min, and the number of times the object was touched was recorded. The object recognition ability (%) was calculated as follows: [familiar object or novel object/(familiar object + novel object)] × 100 [26].

### 2.6. Morris Water Maze Test

The Morris water maze test was carried out for 4 days according to the method described by Morris [27]. The apparatus consists of a circular water pool divided into four quadrants, with four different visual clues (labeled target, circle, triangle, and square). A hidden platform was set under water at about 1 cm in the middle of the target quadrant. The pool was filled with water and a non-toxic white pigment was added. The pool water temperature was maintained at 22 ± 2 °C. The test included three trials per day, with an interval of 4 h between each trial. The first three days were training days in which each mouse swam for 60 s to find the hidden platform and memorize the place. The mouse was allowed to stay on the hidden platform for 15 s, if they arrived at the platform. If not, it was gently guided to the hidden platform and allowed to stay for 15 s. On the final day (testing day), the first trial had the same arrangement as the training days. In the second trial, each mouse was allowed to swim in the pool without the hidden platform for 60 s, and the latency time in the target quadrant was recorded. The percentage of time spent in the target quadrant was expressed as the occupancy of the target quadrant (%). The last trial was performed in transparent water, and the platform was exposed and visible. The time for each mouse to swim and arrive at the exposed platform was recorded.

### 2.7. Test for Aspartate Transaminase (AST) and Alanine Transaminase (ALT)

After completing the behavioral tests, the mice were sacrificed. Blood was collected and centrifuged at 3000 rpm for 15 min to obtain the blood serum [28]. Blood tests for AST and ALT were conducted using AST/glutamate oxaloacetate transaminase (AST/GOT) and ALT/glutamate pyruvate transaminase (ALT/GPT) kits from Asan Pharm (Seoul, Korea).

### 2.8. Western Blotting

The brains were collected and lysed in lysis buffer, a mixture with radioimmunoprecipitation assay buffer (Daejeon, Korea) and 1× protease inhibitor cocktail (Millipore, MA, USA). The supernatant was used to determine the protein concentration using a Bio-Rad protein assay kit (Bio-Rad, Hercules, CA, USA). The quantified protein samples were loaded and separated with 10 or 13% sodium dodecyl sulfate polyacrylamide gels at 90 V for 2 h. Subsequently, the separated proteins were transferred onto polyvinylidene fluoride membranes (Millipore, Burlington, MA, USA) and blocked with 5% skim milk for 1 h. Membranes were incubated with the following primary antibodies: APP (1:1000; A8717; Sigma–Aldrich, St. Louis, MO, USA), BACE (1:1000; #5606; Cell Signaling, Danvers, MA, USA), PS1 (1:1000; Cell Signaling, Danvers, MA, USA), PS2 (1:1000; Cell Signaling, Danvers, MA, USA), and beta-actin (1:1000; #8457; Cell Signaling, Danvers, MA, USA). After overnight incubation at 4 °C, membranes were incubated with secondary anti-rabbit IgG (1:1000; #7074; Cell Signaling, Danvers, MA, USA) at room temperature for 1 h. The proteins were detected using a chemiluminescence detection system (Davinch-Chemi, Seoul, Korea), and protein bands were analyzed using ImageJ software.

### 2.9. Statistical Analyses

All data are expressed as mean ± SD and were analyzed using one-way ANOVA of the Statistical Package for the Social Sciences (SPSS, Chicago, IL, USA) program followed by Duncan’s post-hoc test for multiple comparisons. Student’s *t*-test was used for two-group comparisons in the T-maze test and novel object recognition test. Statistical significance was set at *p* < 0.05.

## 3. Results

### 3.1. Effect of CT on Body Weight Change and Liver Function in Aβ_25-35_-Infused Mice

**Figure 2:**
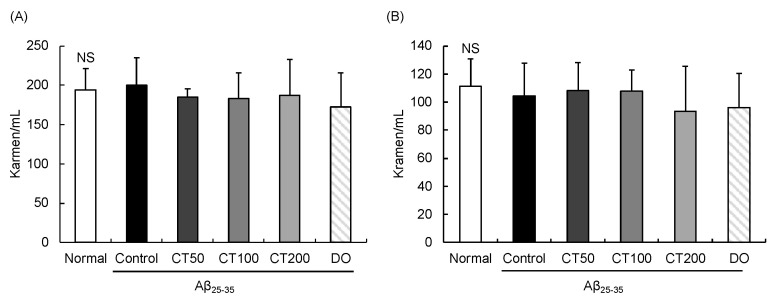
Effect of combination with *Carthamus tinctorius* L. seed and *Taraxacum coreanum* (CT) on AST (**A**) and ALT (**B**) in the serum of Aβ_25-35_ injected mouse. Values are mean ± SD. NS: Non-significance. Normal: 0.9% NaCl + water; Control: Aβ_25-35_ + water; CT50: Aβ_25-35_ + CT (50 mg/kg/day); CT100: Aβ_25-35_ + CT (100 mg/kg/day); CT200: Aβ_25-35_ + CT (200 mg/kg/day); DO: Aβ_25-35_ + donepezil (5 mg/kg/day).

Body weight was recorded five times during the experiment (Table 1). The body weight of the mice on the stocked day were not significantly different among the groups. The body weight was measured before ICV injection, oral administration, behavioral tests, and dissection. The change in body weight of the mice from the beginning to the end of the experiment showed no significant difference among all groups. In addition, the levels of AST and ALT in all the experimental groups showed no significant differences (Figure 2), indicating that CT did not show liver toxicity in mice.

### 3.2. Improvement of Aβ_25-35_-Induced Spatial Impairment in T-Maze Test in CT-Treated Mice

**Figure 3:**
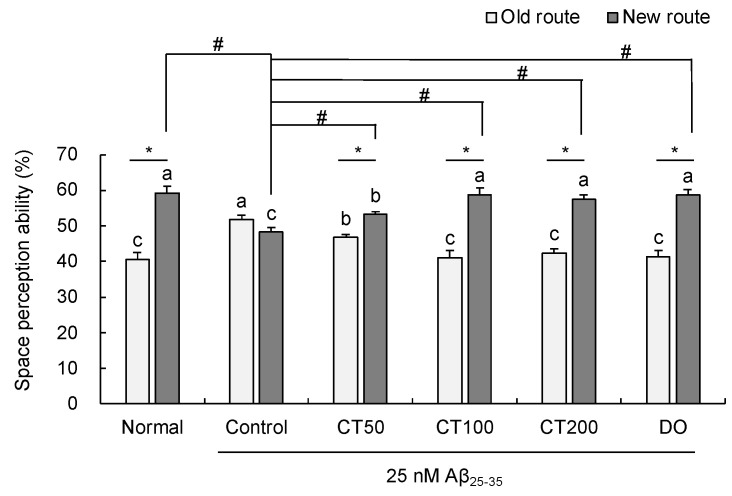
Effect of combination with *Carthamus tinctorius* L. seed and *Taraxacum coreanum* (CT) on T-maze test. Values are mean ± SD. ^a–c^ Means indicated with different letters are significantly different by Duncan’s multiple range test; * *p* < 0.05 vs. old route, # *p* < 0.05 vs. control group. Normal: 0.9% NaCl + water; Control: Aβ_25-35_ + water; CT50: Aβ_25-35_ + CT (50 mg/kg/day); CT100: Aβ_25-35_ + CT (100 mg/kg/day); CT200: Aβ_25-35_ + CT (200 mg/kg/day); DO: Aβ_25-35_ + donepezil (5 mg/kg/day).

In the T-maze test, Aβ_25-35_-injected control mice showed the same performance in the old and new routes, showing Aβ_25-35_-induced cognitive dysfunction (Figure 3). However, after administration of CT (50, 100, and 200 mg/kg), the number of new route entries increased by 53.19%, 58.79%, and 57.59% compared to the old route entry at 46.80%, 41.20%, and 42.41%, respectively. These results indicated that CT prevented Aβ_25-35_-induced spatial memory dysfunction.

### 3.3. Improvement of Aβ_25-35_-Induced Object Recognition Impairment in Novel Object Recognition Test in CT-Treated Mice

**Figure 4:**
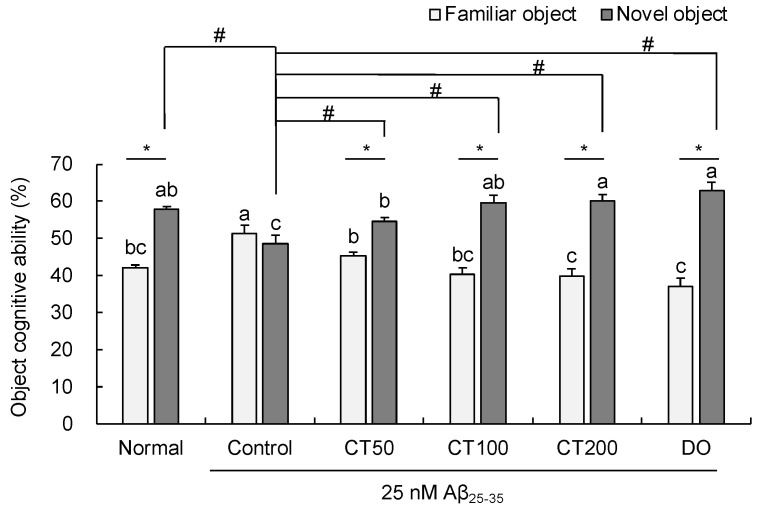
Effect of combination with *Carthamus tinctorius* L. seed and *Taraxacum coreanum* (CT) on novel object recognition test. Values are mean ± SD. ^a–c^ Means indicated with different letters are significantly different by Duncan’s multiple range test; * *p* < 0.05 vs. familiar object, # *p* < 0.05 vs. control group. Normal: 0.9% NaCl + water; Control: Aβ_25-35_ + water; CT50: Aβ_25-35_ + CT (50 mg/kg/day); CT100: Aβ_25-35_ + CT (100 mg/kg/day); CT200: Aβ_25-35_ + CT (200 mg/kg/day); DO: Aβ_25-35_ + donepezil (5 mg/kg/day).

The novel object recognition test explored whether mice were curious about a novel object. No significant differences in the number of touches during exploration of two familiar objects were observed among the groups on the training day (Figure 4). However, after replacing the novel object, the Aβ_25-35_-injected control group showed no significant changes compared to the normal group. However, the object cognitive ability (%) of CT50 (54.73%), CT100 (59.71%), and CT200 (60.11%) was significantly improved compared to that of the control group (48.66%). These results indicate that CT protected against cognitive impairment in Aβ_25-35_-injected mice.

### 3.4. Improvement of Aβ_25-35_-Induced Long-Term Spatial Memory Impairment in Morris Water Maze Test in CT-Treated Mice

**Figure 5:**
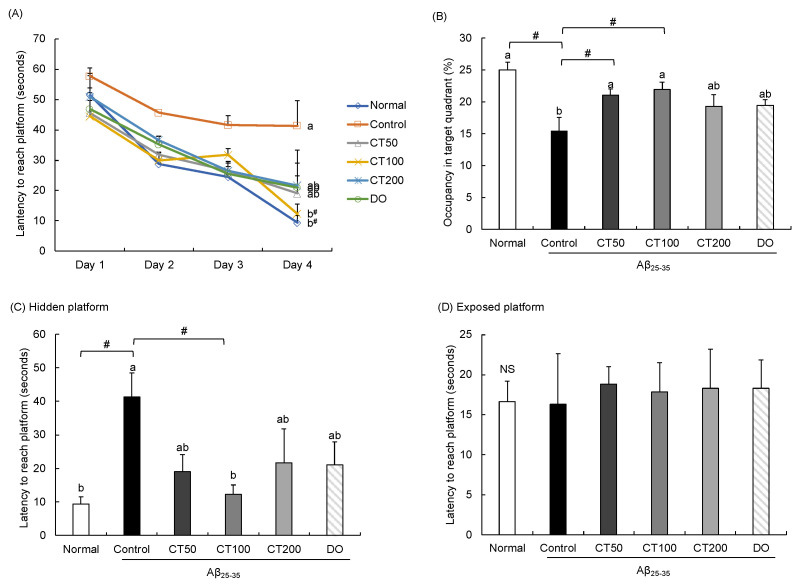
Effect of combination of *Carthamus tinctorius* L. seed and *Taraxacum coreanum* (CT) on Morris water maze test. (**A**) Escape latency to the platform, (**B**) Occupancy time of the target quadrant, (**C**) Latency to reach the hidden platform on the final test day, and (**D**) Latency to reach the exposed platform. Values are mean ± SD. ^a,b^ Means indicated with different letters are significantly different by Duncan’s multiple range test; # *p* < 0.05 vs. control group. NS: Non-significance. Normal: 0.9% NaCl + water; Control: Aβ_25-35_ + water; CT50: Aβ_25-35_ + CT (50 mg/kg/day); CT100: Aβ_25-35_ + CT (100 mg/kg/day); CT200: Aβ_25-35_ + CT (200 mg/kg/day); DO: Aβ_25-35_ + donepezil (5 mg/kg/day).

The results of the Morris water maze test are shown in Figure 5. After 3 days of training, on the fourth day, the escape latency of mice in the control group was longer than that of mice in the normal group. Moreover, the groups administered with CT had significantly shortened escape latency compared to the control group (Figure 5A). In the probe trial, compared with the normal group, the time spent in exploring the target quadrant in the control group was decreased; however, the time spent in the target quadrant was significantly increased in the CT group. In particular, the CT100 group spent a significantly longer time in the target quadrant than the other sample groups (Figure 5B). In another probe trial, the water was transparent and the mice were placed in water to reach the exposed platform. No significant differences were observed among the groups, indicating that the ability of the mice to reach the platform was not related to their vision or physical strength (Figure 5C,D). These results suggest that CT treatment could protect against cognitive impairment and improve memory dysfunction.

### 3.5. Regulatory Effect of CT on Amyloidogenic Pathway in Aβ_25-35_-Infused Mice

**Figure 6:**
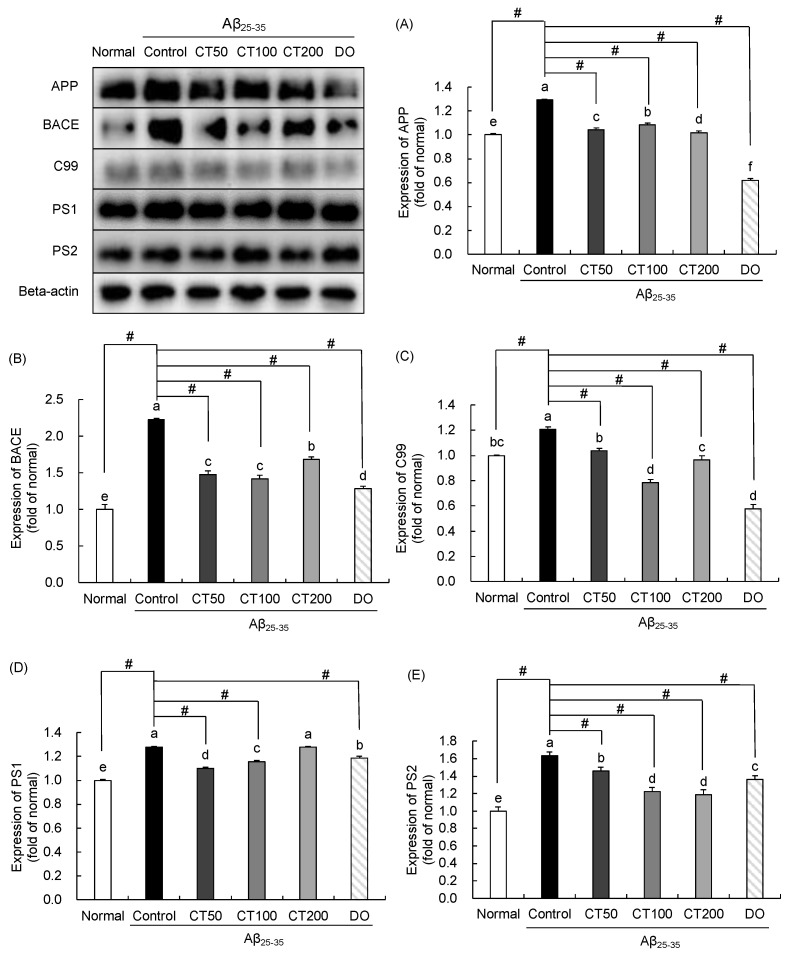
Effect of combination of *Carthamus tinctorius* L. seed and *Taraxacum coreanum* (CT) on APP processing-associated protein expression. Expression of APP (**A**), BACE (**B**), C99 (**C**), PS1 (**D**), and PS2 (**E**). Values are mean ± SD. ^a–f^ Means indicated with different letters are significantly different by Duncan’s multiple range test; # *p* < 0.05 vs. control group. Normal: 0.9% NaCl + water; Control: Aβ_25-35_ + water; CT50: Aβ_25-35_ + CT (50 mg/kg/day); CT100: Aβ_25-35_ + CT (100 mg/kg/day); CT200: Aβ_25-35_ + CT (200 mg/kg/day); DO: Aβ_25-35_ + donepezil (5 mg/kg/day).

To understand the protective mechanism of CT against cognitive impairment, several factors related to the amyloidogenic pathway were analyzed by Western blotting. As shown in Figure 6A–E, following injection of Aβ_25-35_ peptides, APP expression in the brain increased, whereas it decreased by the administration of CT. BACE-1 is an enzyme that cleaves APP and promotes the production of Aβ. Increased levels of BACE was observed in the Aβ_25-35_-injected group, and decreased levels were observed in the normal and CT groups. BACE generates the C99 fragment, which releases Aβ by regulating PS1 and PS2. The protein expression of C99, PS1, and PS2 was increased in the Aβ_25-35_-injected control group compared with that in the normal group; however, it decreased after treatment with CT. These results indicate that the administration of CT could inhibit the generation of Aβ. 

## 4. Discussion

*C. tinctorius* L. seeds have been reported to improve cognitive dysfunction in vivo [29], showing reduced oxidative stress and improved cognitive dysfunction in chronic alcohol-induced mice. *C. tinctorius* L. seed inhibited the activity of AChE induced by scopolamine injection and attenuated memory and learning disabilities in behavioral tests [30]. According to the previous studies, phenolic compounds, such as *N*-ferulyolserotonin, *N*-(*p*-coumaroyl)serotonin, 2-hydroxyarctiin, and acacetin, have been isolated and identified from *C. tinctorius* L. seed [31,32]. In addition, the serotonin derivatives, *N*-ferulyolserotonin and *N*-(*p*-coumaroyl)serotonin have been identified as the major phenolic components in the ethanol-ethyl acetate extract of *C. tinctorius* L. seed [33]. In addition, *N*-(*p*-coumaroyl)serotonin, and *N*-feruloylserotonin have been reported to prevent high glucose-induced toxicity in PC12 neuronal cells [34]. 

*T. coreanum* has been used as an edible herbal medicine. The ethyl acetate fraction of *T. coreanum* attenuated cognitive dysfunction in Aβ-induced AD mice and reduced lipid peroxidation and nitric oxide production [20]. It has been reported that *T. coreanum* prevents glutamate-induced oxidative stress in hippocampal neuronal cells, showing a neuroprotective effect by regulating the HO-1/Nrf2 pathway [35]. On the other hand, phytochemical constituents have been isolated from *T. coreanum* including flavones (luteolin, luteolin-7-glucoside, etc.) and hydroxycinnamic acid derivatives (caffeic acid, chlorogenic acid, 1-caffeoylglycerol, chicoric acid, etc.) [36,37]. Among them, chicoric acid has been identified as the major component of *T. coreanum*, followed by chlorogenic acid [37]. Chicoric acid showed inhibitory effects against LPS-induced memory dysfunction and amyloidogenesis in mice [38]. 

In recent years, the concept of multitarget therapy using herbal mixtures for disease treatment has received extensive attention. Herbal mixtures are known to have greater efficacy than a single herb; thus, they have been applied in clinical and pharmacological research. Moreover, the use of herbal mixtures can also lower the dosage in comparison with single plant extracts, which are usually used at high doses [39]. Herbal mixtures, which are marked by multiple components that affect multiple pathways, have become popular in the research of neurodegenerative disease. An increasing number of studies have indicated beneficial roles of herbal combination on neuroprotection. For instance, a herbal pair, *Alpinia oxyphylla* and *Schisandra chinensis*, as well as their major compounds, schisandrin and nootkatone, have demonstrated a shorter escape latency time in the Morris water maze and an inhibitory effect on inflammatory response via TLR4/NF-ĸB/NLRP3 pathway in amyloid beta (Aβ)-induced mouse model of Alzheimer’s disease [40]. Moreover, the *Epimedii folium*—*Curculiginis rhizome* pair has been reported to reduce oxidative stress by decreasing the level of malondialdehyde and increasing the activities of superoxide dismutase, catalase, and glutathione in Aβ-injected rats [41]. These results demonstrated that herbal mixtures could attenuate Aβ-induced neuronal damage by regulation of various pathways. Our previous study has demonstrated a synergy neuroprotective effect of the combination with *C. tinctorius* L. seed and *T. coreanum* at a ratio of 5:5 compared to the individual treatment of *C. tinctorius* L. seed or *T. coreanum*. The results showed that in the group treated *C. tinctorius* L. seed or *T. coreanum* alone increased the number of entries in the new route compared with the old route on T-maze test in Aβ-infused mice. However, the combination of *C. tinctorius* L. seed and *T. coreanum* synergistically enhanced the entries to the new route, which was significantly higher than the treatment of *C. tinctorius* L. seed or *T. coreanum*. Moreover, in the protein expression of insulin-degrading enzyme, which is related to Aβ clearance, the group treated by the combination at 5:5 ratio significantly increased more than the group treated with *C. tinctorius* L. seed or *T. coreanum* alone. Our results indicated that the combination of *C. tinctorius* L. seed and *T. coreanum* exerts a protective effect against Aβ-infused mice by improving spatial recognitive ability and increasing Aβ clearance-related protein level. Therefore, we considered that the combination of *C. tinctorius* L. seed and *T. coreanum* exerts higher neuroprotective effect than single extract of *C. tinctorius* L. seed or *T. coreanum* [21]. Therefore, we hypothesized that CT at a ratio of 5:5 exerts a protective effect on cognitive impairment and could be a functional material that can play a role in protection of AD pathologies. However, the effects of different concentrations of CT on cognitive dysfunction have not yet been investigated. Therefore, we used CT at a ratio of 1:1 with three different concentrations (low, medium, and high = 50, 100, and 200 mg) to explore the protective activity in a mouse model of cognitive impairment. 

In this study, learning and memory abilities were measured using the T-maze, novel object recognition, and Morris water maze tests. The results showed spatial cognitive dysfunction in the T-maze test and object recognition disorder in the novel object recognition test, after injection of Aβ_25-35_ in the control group. However, treatment with different concentrations of CT significantly improved cognitive function in Aβ_25-35_-injected mice. In particular, treatment with 100 and 200 mg/kg dose in the T-maze test showed higher space perception ability. These results indicate that CT protects against Aβ_25-35_-induced memory dysfunction.

The Morris water maze test is a type of reference memory task that has been developed for rodents to find a hidden platform by using visual cues [42]. During the three days of training, mice that were administered CT at all concentrations markedly showed a decrease in the latency time, while it remained stable in the Aβ_25-35_-injected control group. The time to reach the exposed platform was not significantly different among the groups. In contrast, time taken to reach the hidden platform was shorter in the CT mixture-treated groups than in the control group. In addition, we observed that the time spent in the target quadrant was increased by CT. These results demonstrate that CT improved the memory function and enhanced learning ability, suggesting a role of CT treatment in improving the reference memory.

We tested for liver toxicity to determine whether treatment with CT would injure the liver. Serum AST and ALT levels are the most common biochemical parameters used to diagnose liver damage [43]. Oral administration of CT at all doses did not show statistically significant differences in these values compared with the normal group, suggesting that CT did not affect liver function.

Several enzymes, including β-secretase and γ-secretase, play critical roles in the production of Aβ [44]. APP regulates protein transportation between cells [45]. However, APP mutations can be induced by Aβ in both tissues and cells [46]. Schmitt et al. reported that APP increased in HL-60 cells after being treated with Aβ_25-35_ [47]. In Aβ_1-42_-injected mouse brain, APP protein expression was markedly higher than that in the normal group [48]. In the present study, APP expression in the Aβ_25-35_-injected control group was significantly higher than that in the normal group, indicating that Aβ increased APP expression, and the administration of CT significantly decreased the protein expression. In addition, BACE exerts β-secretase activity in the brain, promoting AD pathology [44]. Neurodegeneration induced by ICV injection of Aβ_1-42_ in mice significantly increased BACE expression [49]. In addition, BACE cleaves APP and subsequently generates sAPPβ and C99 [50]. C99 is further cleaved by γ-secretase, which has four components: presenilin, nicastrin, presenilin enhancer, and anterior pharynx defective 1. Presenilin (PS1 and PS2) effectively performs the catalytic role of γ-secretase and promotes the production of Aβ [51]. Several studies have shown that Aβ-induced cognitive deficits significantly increased C99 levels compared with non-injected rats, and enhanced expression of presenilin was detected in Aβ-injected APP/PS1 Tg mice [52,53]. In the present study, the results show increased protein levels of BACE, C99, PS1, and PS2 in the Aβ_25-35_-injected control group, but decreased levels after administration of CT. Therefore, CT regulates APP processing by reducing APP protein expression and inhibiting the activity of the APP processing-related enzyme.

## 5. Conclusions

In conclusion, our results show that ICV injection of Aβ_25-35_ effectively induces cognitive dysfunction and reference memory deficits in mice, which were improved by the administration of CT. In addition, CT effectively inhibited the amyloidogenic pathway by downregulating APP processing-associated protein expression. This study suggests that CT might be a functional food candidate for AD prevention.

## Figures and Tables

**Table 1:** Effect of combination with *Carthamus tinctorius* L. seed and *Taraxacum coreanum* (CT) on body weight and body weight gain against Aβ_25-35_-injected mouse.

Groups	Body Weight (g)					
	Stocked Day	Injection	Intragastric Administration	Behavioral Experiment	Dissection	Body Weight Gain
Normal	27.4 ± 1.3 ^NS^	32.3 ± 1.3 ^NS^	33.4 ± 1.1 ^NS^	33.6 ± 1.6 ^NS^	33.8 ± 1.3 ^NS^	6.4 ± 0.6 ^NS^
Control	27.6 ± 1.1	32.0 ± 0.3	33.4 ± 1.0	34.6 ± 1.6	34.2 ± 1.4	6.6 ± 0.5
CT50	27.1 ± 1.1	32.1 ± 0.9	33.0 ± 1.1	33.6 ± 1.5	33.0 ± 1.6	6.0 ± 1.2
CT100	27.5 ± 0.6	33.2 ± 1.1	33.9 ± 1.0	34.5 ± 1.7	33.5 ± 1.1	6.0 ± 1.1
CT200	27.6 ± 1.3	32.4 ± 1.4	33.7 ± 1.6	33.7 ± 1.7	33.5 ± 2.2	5.9 ± 1.4
DO	26.8 ± 0.8	32.8 ± 0.8	32.8 ± 2.1	33.0 ± 1.2	32.3 ± 1.0	5.5 ± 1.1

Values are mean ± SD. ^NS^: Non-significance. Normal: 0.9% NaCl + water; Control: Aβ_25-35_ + water; CT50: Aβ_25-35_ + CT (50 mg/kg/day); CT100: Aβ_25-35_ + CT (100 mg/kg/day); CT200: Aβ_25-35_ + CT (200 mg/kg/day); DO: Aβ_25-35_ + donepezil (5 mg/kg/day).

## Data Availability

The data associated with this research are available and can be obtained by contacting the corresponding author.
